# Supplementary material for: IL-25 blockade augments antiviral immunity during respiratory virus infection
Source: Commun Biol. 2022 May 4;5:415. doi: 10.1038/s42003-022-03367-z (PMC9068710; doi:10.1038/s42003-022-03367-z)
Supplement: Supplementary file 2 — Supplementary Information [file 42003_2022_3367_MOESM2_ESM.pdf]

IL-25 blockade augments antiviral immunity during respiratory virus infection

Teresa C. Williams[1], Su-Ling Loo[1], Kristy S. Nichol[1], Andrew T. Reid[1], Punnam C. Veerati[1], Camille Esneau[1], Peter A.B. Wark[1,2], Christopher L. Grainge[1,2], Darryl A. Knight[1,3,4], Thomas Vincent[5], Crystal L. Jackson[5], Kirby Alton[5], Richard A. Shimkets[5], Jason L. Girkin[1] and Nathan W. Bartlett[1]

SUPPLEMENTARY MATERIALS

Supplementary Figures:

a

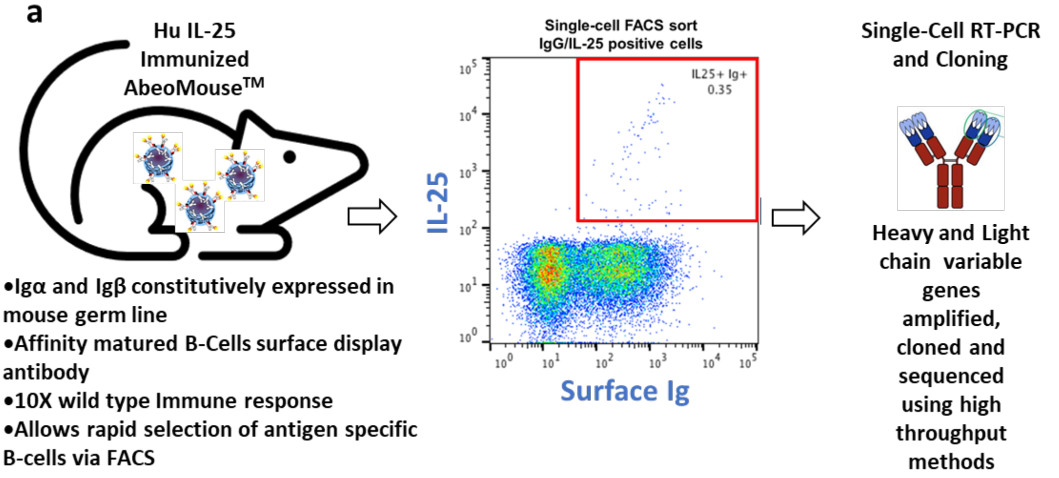

LNR125 Potency IC$_{50}$ in HT29 Cell Assay (ug/mL)			LNR125 Affinity SPR (KD)	
Human	Human	Mouse	Human	Mouse
(E. coli)	(HEK)	(NSO)	(HEK)	(NSO)
0.3	0.075	0.15	5.3pM	7.3pM

b

2° by row / Coating by column	125	126	hIL-25
125-HRP	0.448	1.717	1.393
126-HRP	1.56	0.53	1.697

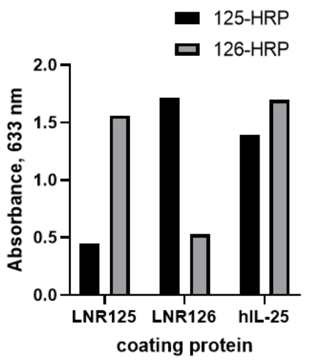

Supplementary Figure 1: Secondary control staining for endoscopic bronchial biopsies and ALI-differentiated BECs. Immunofluorescence staining of anti-IL-25 and anti-IL-17RB secondary antibodies in (a) endoscopic biopsies and (b) ALI-BECs to confirm the specificity of secondary antibodies. Representative of n=5 biopsies. Scale bars represent 20µm and 25µm respectively, as indicated.

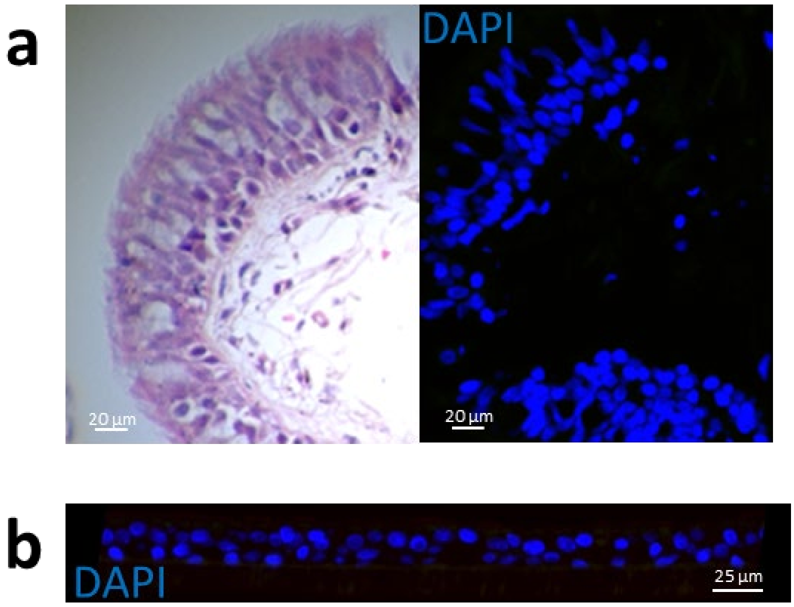

Supplementary Figure 2: LNR125 treatment increased antiviral gene expression. RNA was harvested 4 d.p.i from n=6 RV-A1 infected ALI-differentiated BECs treated with LNR125 or LNR2. Isolated RNA underwent transcriptomic analysis. (a-b) Differentially expressed anti-viral genes. (c) Immunoblot validation and densitometry of antiviral genes, representative blot. Bars are representative of median with IQR analysed by Wilcoxon matched-pairs t-test. light grey and dark grey indicate LNR2 and LNR125 treatment, respectively. * $P < 0.05$, ns = not significant.

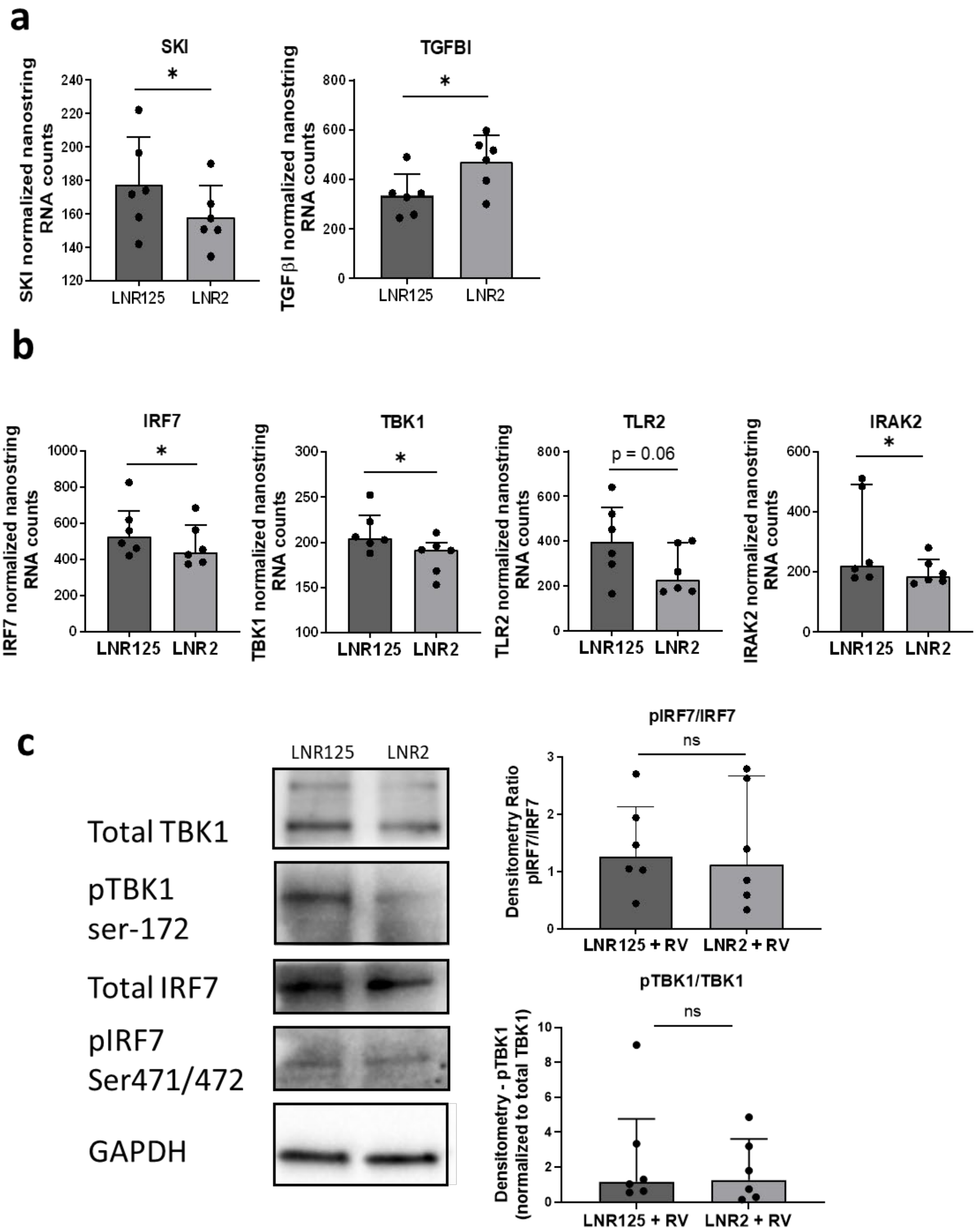

Supplementary Figure 3: Isolation and characterization of LNR125 and epitope binding to confirm that that LNR125 and LNR126 bind to distinct epitopes (a) AbeoMouse™ has been engineered to constitutively express multiple genes resulting in a hyper immune response and surface antibody expression during all stages of B cell differentiation. This enables the generation of therapeutic mAbs through the selection and sorting of antigen specific B-cells

producing the most affinity matured antibodies. Using this technology, a potent mAb (LNR125) with low picomolar binding affinity for both human and mouse IL-25 was isolated. **ABM125/LNR125 was developed by Abeome Corporation now Lanier Biotherapeutics** (b) Paired sandwich ELISA alternating coating antibodies (LNR125 and LNR126) or recombinant human IL-25 (hIL-25)- and 2° (125-HRP and 126-HRP) antibodies demonstrate that LNR125 and LNR126 belong to different epitope bins.

Supplementary Tables:

Supplementary Table 1: Clinical Characteristics of differentiated BEC donors

	Healthy	Asthma
number, n	2	14
Age, years (SD)	63.5 (16.2)	56.7 (13.7)
Gender (M/F)	(0/2)	(4/10)
Atopy (SPT positive)	1	9
Severity	NA	Mild (3)
	NA	Moderate (2)
	NA	Severe (9)
Eosinophil counts (% total sputum cells) (SD)	0.13 (0.2)	5.79 (10.9)
FEV1, % predicted (SD)	80 (9.8)	77 (19.7)
FVC, % predicted (SD)	86.5 (7.8)	86.5 (10.3)
Daily ICS dose, beclomethasone equivalent, ug (SD)	NA	257 (90.1)

FEV1, Forced expiratory volume 1 s; FVC, Forced vital capacity; ICS, Inhaled corticosteroid; SPT, skin prick test

Supplementary Table 2. Nucleotide sequences of TaqMan primers and probes

Gene	Forward	Reverse	Probe
18S	CGCCGCTAGAGGTGAAATTCT	CATTCTTGGCAAATGCTTTCG	FAMACCGGCGCAAGACGGACCAGA-TAMRA
IL-25	GAGATATGAGTTGGACAGAGACTTGAA	CCATGTGGGAGCCTGTCTGTA	FAMCTCCCCCAGGACCTGTACCACGC-TAMRA
IL-17RB	GCTCTTATCCAACACAGCACTA	CACCGTAGCACCTTCACTATC	FAMAGCCACACCAGAAGAAACAAACGC-TAMRA
RV-A1	GTGAAGAGCCsCrTGTGCT	GCTsCAGGGTTAAGGTTAGCC	FAMTGAGTCCTCCGGCCCCTGAATG-TAMRA
229E	CAGTCAAATGGGCTGATGCA	AAAGGGCTATAAAGAGAATAAGGTATTCT	FAMCCCTGACGACCACGTTGTGGTTCA-TAMRA
IFN-β	CGCCGCATTGACCATCTA	TTAGCCAGGAGGTTCTCAACAATAGTCTCA	FAMTCAGACAAGATTCATCTAGCACTGGCTGGA-TAMRA
IFN-λ2/3	CTGCCACATAGCCCAGTTCA	AGAAGCGACTCTTCTAAGGCATCTT	FAMTCTCCACAGGAGCTGCAGGCCTTTA-TAMRA

All sequences presented in 5’ to 3’ orientation.
