# Supplementary material for: IL-25 blockade augments antiviral immunity during respiratory virus infection
Source: Commun Biol. 2022 May 4;5:415. doi: 10.1038/s42003-022-03367-z (PMC9068710; doi:10.1038/s42003-022-03367-z)
Supplement: Supplementary file 3 — Description of Additional Supplementary Files [file 42003_2022_3367_MOESM3_ESM.pdf]

Description of Additional Supplementary Files

File name: Supplementary Data 1
Description: Dataset corresponding to all primary data presented in article figures.
