# Supplementary material for: IL-25 blockade augments antiviral immunity during respiratory virus infection
Source: Commun Biol. 2022 May 4;5:415. doi: 10.1038/s42003-022-03367-z (PMC9068710; doi:10.1038/s42003-022-03367-z)
Supplement: Supplementary file 5 — NR Reporting Summary [file 42003_2022_3367_MOESM5_ESM.pdf]

nature portfolio

Corresponding author(s): *Double-blind peer review submissions: write DBPR and your manuscript number here instead of author names.*

Last updated by author(s): *YYYY-MM-DD*

Reporting Summary

Nature Portfolio wishes to improve the reproducibility of the work that we publish. This form provides structure for consistency and transparency in reporting. For further information on Nature Portfolio policies, see our Editorial Policies and the Editorial Policy Checklist.

Statistics

For all statistical analyses, confirm that the following items are present in the figure legend, table legend, main text, or Methods section.

n/a	Confirmed	
☐	☒	The exact sample size (n) for each experimental group/condition, given as a discrete number and unit of measurement
☐	☒	A statement on whether measurements were taken from distinct samples or whether the same sample was measured repeatedly
☐	☒	The statistical test(s) used AND whether they are one- or two-sided<br>*Only common tests should be described solely by name; describe more complex techniques in the Methods section.*
☒	☐	A description of all covariates tested
☐	☒	A description of any assumptions or corrections, such as tests of normality and adjustment for multiple comparisons
☐	☒	A full description of the statistical parameters including central tendency (e.g. means) or other basic estimates (e.g. regression coefficient) AND variation (e.g. standard deviation) or associated estimates of uncertainty (e.g. confidence intervals)
☐	☒	For null hypothesis testing, the test statistic (e.g. F, t, r) with confidence intervals, effect sizes, degrees of freedom and P value noted<br>*Give P values as exact values whenever suitable.*
☒	☐	For Bayesian analysis, information on the choice of priors and Markov chain Monte Carlo settings
☒	☐	For hierarchical and complex designs, identification of the appropriate level for tests and full reporting of outcomes
☒	☐	Estimates of effect sizes (e.g. Cohen's d, Pearson's r), indicating how they were calculated

Our web collection on statistics for biologists contains articles on many of the points above.

Software and code

Policy information about availability of computer code

Data collection	No software was used.
Data analysis	Analysis was performed using GraphPad Prism v8.3 software, as outlined in the Methods section.

For manuscripts utilizing custom algorithms or software that are central to the research but not yet described in published literature, software must be made available to editors and reviewers. We strongly encourage code deposition in a community repository (e.g. GitHub). See the Nature Portfolio guidelines for submitting code & software for further information.

Data

Policy information about availability of data

All manuscripts must include a data availability statement. This statement should provide the following information, where applicable:

- Accession codes, unique identifiers, or web links for publicly available datasets
- A description of any restrictions on data availability
- For clinical datasets or third party data, please ensure that the statement adheres to our policy

All data is provided in manuscript file.

Field-specific reporting

Please select the one below that is the best fit for your research. If you are not sure, read the appropriate sections before making your selection.

☒ Life sciences ☐ Behavioural & social sciences ☐ Ecological, evolutionary & environmental sciences

For a reference copy of the document with all sections, see nature.com/documents/nr-reporting-summary-flat.pdf

Life sciences study design

All studies must disclose on these points even when the disclosure is negative.

Sample size	Sample sizes were determined based on previous experience with the described infection model. No specific sample size calculation was performed.
Data exclusions	No data were excluded from analysis.
Replication	Biological replicates were performed as indicated in the figure legends.
Randomization	Allocation was random.
Blinding	Blinding to treatment was not possible.

Reporting for specific materials, systems and methods

We require information from authors about some types of materials, experimental systems and methods used in many studies. Here, indicate whether each material, system or method listed is relevant to your study. If you are not sure if a list item applies to your research, read the appropriate section before selecting a response.

Materials & experimental systems

n/a	Involved in the study	
☐	☒	Antibodies
☒	☐	Eukaryotic cell lines
☒	☐	Palaeontology and archaeology
☐	☒	Animals and other organisms
☐	☒	Human research participants
☐	☒	Clinical data
☒	☐	Dual use research of concern

Methods

n/a	Involved in the study	
☒	☐	ChIP-seq
☐	☒	Flow cytometry
☒	☐	MRI-based neuroimaging

Antibodies

Antibodies used	All information provided in manuscript Methods section, including antibodies for ELISA, in vivo treatment and immunofluorescence.
Validation	Assessment of experimental blocking antibodies is described in the manuscript, including Supplementary Figure 1. Specificity of commercial antibodies was per manufacturers documentation.

Animals and other organisms

Policy information about studies involving animals; ARRIVE guidelines recommended for reporting animal research

Laboratory animals	6–8-week-old, wild-type, female BALB/c mice were obtained from Australian Bioresources (ABR, Moss Vale, NSW)
Wild animals	N/A
Field-collected samples	N/A
Ethics oversight	Human samples approved via University of Newcastle and Hunter New England Area Health ethics committee (05/08/10/3.09); Mouse studies per protocol A-2016-605, reviewed and approved by the University of Newcastle Animal Care and Ethics Committee.

Note that full information on the approval of the study protocol must also be provided in the manuscript.

Human research participants

Policy information about studies involving human research participants

Population characteristics	Endobronchial biopsies obtained from healthy donors and patients with asthma, as described in manuscript.
Recruitment	Recruitment of volunteers via John Hunter Hospital Respiratory Department and Hunter Medical Research Institute.
Ethics oversight	Human samples approved via University of Newcastle and Hunter New England Area Health ethics committee (05/08/10/3.09)

Note that full information on the approval of the study protocol must also be provided in the manuscript.

Clinical data

Policy information about clinical studies

All manuscripts should comply with the ICMJE guidelines for publication of clinical research and a completed CONSORT checklist must be included with all submissions.

Clinical trial registration	N/A
Study protocol	N/A
Data collection	N/A
Outcomes	N/A

Flow Cytometry

Plots

Confirm that:

☒ The axis labels state the marker and fluorochrome used (e.g. CD4-FITC).

☒ The axis scales are clearly visible. Include numbers along axes only for bottom left plot of group (a 'group' is an analysis of identical markers).

☒ All plots are contour plots with outliers or pseudocolor plots.

☒ A numerical value for number of cells or percentage (with statistics) is provided.

Methodology

Sample preparation	Detail provided in manuscript; cytokine / chemokine quantification from culture supernatants
Instrument	FACS Canto II (Beckman Coulter)
Software	LEGENDplex v8.0 (Biolegend)
Cell population abundance	N/A
Gating strategy	Per manufacturer's instructions - LEGENDplex human anti-virus response multiplex flow cytometry panel

☐ Tick this box to confirm that a figure exemplifying the gating strategy is provided in the Supplementary Information.
